# Childhood adversity and clinical and psychosocial outcomes in psychosis

**DOI:** 10.1017/S2045796019000684

**Published:** 2019-12-16

**Authors:** S. Turner, C. Harvey, L. Hayes, D. Castle, C. Galletly, S. Sweeney, S. Shah, L. Keogh, M. J. Spittal

**Affiliations:** 1 Quality and Service Improvement, NorthWestern Mental Health, 300 Grattan St, Parkville, Victoria 3050, Australia; 2 Department of Psychiatry, The University of Melbourne, Parkville, Victoria 3010, Australia; 3 Psychosocial Research Centre, NorthWestern Mental Health, 130 Bell St, Coburg, Victoria 3058, Australia; 4 Parenting Research Centre, 232 Victoria Parade, East Melbourne, Victoria 3002, Australia; 5 St Vincent's Hospital, 41 Victoria Parade, Fitzroy, Victoria 3065, Australia; 6 Discipline of Psychiatry, University of Adelaide, Adelaide, South Australia 5005, Australia; 7 Ramsay Health Care (SA) Mental Health Services, 33 Park Tce, Gilbertson, South Australia 5081, Australia; 8 North Adelaide Local Health Network, Ward 1G, Lyell McEwin Hospital, Haydown Road, Elizabeth Vale, South Australia 5112, Australia; 9 Neuropsychiatric Epidemiology Research Unit, School of Psychiatry and Clinical Neurosciences, The University of Western Australia, 35 Stirling Highway, Crawley, Western Australia 6009, Australia; 10 Melbourne School of Population and Global Health, The University of Melbourne, Parkville, Victoria 3010, Australia

**Keywords:** Adverse life events, child abuse, childhood sexual abuse, childhood trauma, psychosis

## Abstract

**Aims:**

Associations between childhood abuse and various psychotic illnesses in adulthood are commonly reported. We aim to examine associations between several reported childhood adverse events (sexual abuse, physical abuse, emotional abuse, neglect and interpersonal loss) among adults with diagnosed psychotic disorders and clinical and psychosocial outcomes.

**Methods:**

Within a large epidemiological study, the 2010 Australian National Survey of Psychosis (Survey of High Impact Psychosis, SHIP), we used logistic regression to model childhood adverse events (any and specific types) on 18 clinical and psychosocial outcomes.

**Results:**

Eighty percent of SHIP participants (1466/1825) reported experiencing adverse events in childhood (sexual abuse, other types of abuse and interpersonal loss). Participants reporting any form of childhood adversity had higher odds for 12/18 outcomes we examined. Significant associations were observed with all psychosocial outcomes (social dysfunction, victimisation, offending and homelessness within the previous 12 months, and definite psychosocial stressor within 12 months of illness onset), with the strongest association for homelessness (odds ratio (OR) = 2.82). Common across all adverse event types was an association with lifetime depression, anxiety and a definite psychosocial stressor within 12 months of illness onset. When adverse event types were non-hierarchically coded, sexual abuse was associated with 11/18 outcomes, other types of abuse 13/18 and, interpersonal loss occurring in the absence of other forms of abuse was associated with fewer of the clinical and psychosocial outcomes, 4/18. When adverse events types were coded hierarchically (to isolate the effect of interpersonal loss in the absence of abuse), interpersonal loss was associated with lower odds of self-reproach (OR = 0.70), negative syndrome (OR = 0.75) and victimisation (OR = 0.82).

**Conclusions:**

Adverse childhood experiences among people with psychosis are common, as are subsequent psychosocial stressors. Mental health professionals should routinely enquire about all types of adversities in this group and provide effective service responses. Childhood abuse, including sexual abuse, may contribute to subsequent adversity, poor psychosocial functioning and complex needs among people with psychosis. Longitudinal research to better understand these relationships is needed, as are studies which evaluate the effectiveness of preventative interventions in high-risk groups.

## Introduction

According to global estimates, 7–36% of women and 3–29% of men report being victims of sexual abuse during childhood (Pereda *et al*., 2009). In Australia, the prevalence is 14–20% for women and 3–10% for men (Moore *et al*., 2010). The evidence increasingly supports strong associations between childhood sexual abuse and psychotic disorders in later life. Retrospective studies show that a high proportion of people with psychotic disorders have previously experienced sexual abuse (Bendall *et al*., 2008; Cutajar *et al*., 2010; Bebbington *et al*., 2011; Mauritz *et al*., 2013). Research has also shown strong associations between schizophrenia, as well as other mental disorders including depression, anxiety, post-traumatic stress disorder, dissociative disorders, substance dependence and self-harm and childhood abuse, including sexual, physical and emotional abuse, and neglect (Van der Kolk *et al*., 2005; Cutajar *et al*., 2010; Gonzalez *et al*., 2012; Varese *et al*., 2012; Matheson *et al*., 2013; van Nierop *et al*., 2015; Palmier-Claus *et al*., 2016). Although it has long been recognised that stressful life events and difficulties contribute to depression and anxiety (see Craig, 2010), less is known about how relatively common adverse experiences (e.g. parental separation) may contribute to psychoses.

Psychosocial outcomes refer to a person's recovery in areas such as vocational and social functioning (Killackey *et al*., 2015; Stolerman and Price, 2015). This is an important but neglected area of research (Fossey and Harvey, 2001; Misiak *et al*., 2017). Poor psychosocial outcomes directly affect mental health consumers and their families as well as contributing to a general impact on quality of life (National Mental Health Commission, 2014; Cotter *et al*., 2015; Poon *et al*., 2017). For these reasons, understanding the antecedents of poor psychosocial outcomes is relevant for consumers and families, clinicians, policymakers and researchers alike (Felitti *et al*., 1998). Improved understanding of psychosocial stressors both preceding and at illness onset also offers an opportunity for prevention and early intervention. In a previous study, we examined the relationship between childhood abuse and psychosis and reported high rates (31%) of any childhood abuse before illness onset (Shah *et al*., 2014), but our investigation mostly focused on clinical and physical health outcomes, rather than psychosocial outcomes and the recurrence of psychosocial stressors.

Research on psychosocial outcomes among people with psychosis has highlighted that individuals with significant abuse histories often have difficulty with employment, housing and social functioning (Gil *et al*., 2009; Alameda *et al*., 2015; Cotter *et al*., 2015). Other studies have drawn a connection between early and subsequent exposure to psychosocial stressors (e.g. victimisation, homelessness, crime and offending) and an increased likelihood of developing a psychotic response (Rosenberg *et al*., 2007; Pluck *et al*., 2011; Lataster *et al*., 2012). These findings are interesting but limited because of a reliance on single questions to assess trauma – usually defined as child abuse (physical and sexual) or sexual abuse only; little consideration of potential confounders (for instance, family history of mental illness and cannabis use); examination of a small number of outcomes and limited power with only a small number of individuals experiencing the exposure or the outcome (Gil *et al*., 2009; Hooven *et al*., 2012; Berthelot *et al*., 2015; Trauelsen *et al*., 2015; Cristobal-Narvaez *et al*., 2016; Gallagher and Jones, 2016; Holshausen *et al*., 2016).

Our aim was to address some of the gaps and limitations in previous studies by examining all forms of abuse and other negative experiences in childhood and their relationships with a range of outcome variables, adjusting for a wide variety of potential confounders. These are: age (Bentall *et al*., 2012), sex (Bentall *et al*., 2012; Matheson *et al*., 2013), currently married or previously married (Bendall *et al*., 2008), presence of children or step children (Bendall *et al*., 2008), born in Australia (Varese *et al*., 2012), highest qualification is a school-level qualification (Bendall *et al*., 2008; Bentall *et al*., 2012; Varese *et al*., 2012), family history of mental illness (Bendall *et al*., 2008; Varese *et al*., 2012; Matheson *et al*., 2013), socioeconomic status of the participant's residence (Bentall *et al*., 2012; Varese *et al*., 2012) and lifetime cannabis use (Janssen *et al*., 2004; Bendall *et al*., 2008; Varese *et al*., 2012). Specifically, we examined the association between exposure to diverse adverse childhood events and a number of mental health symptoms and diagnoses (Bendall *et al*., 2008; Varese *et al*., 2012; Van Nierop *et al*., 2015) as well as important psychosocial outcomes (Rosenberg *et al*., 2007; Gilbert *et al*., 2009; Alameda *et al*., 2015). Our primary hypothesis was that any adverse event in childhood (defined as sexual, physical and emotional abuse and neglect, and interpersonal loss) would be associated with clinical and psychosocial impairment. Our secondary hypothesis was that different types of abuse as well as interpersonal loss would also be associated with impairment.

## Method

### Overview

This study analysed data collected from the 2010 Australian National Survey of Psychosis (Survey of High Impact Psychosis, SHIP) (Morgan *et al*., 2012, 2014). The survey methodology is described in detail elsewhere (Morgan *et al*., 2012; Shah *et al*., 2014). A cross-sectional survey was conducted across seven catchment sites in five states. Because psychosis is comparatively rare, sampling was done in two stages. A census of people with psychosis was taken from three mutually exclusive groups: people in contact with public mental health services during the census month (March 2010); people not in contact with those services, but in contact with non-government organisations supporting people with mental illness during the census month and people not in contact with either of these two services during the census month but in contact with public mental health services during the preceding 11 months. All those identified in the census were screened using the Psychosis Screen (Jablensky *et al*., 2000) and 7955 screened positive. From this, a random sample (*n*  =  1825) stratified by age (18–34 years and 35–65 years) were selected and interviewed by trained mental health professionals. The response rate was 44% and there was no evidence of selection bias (Morgan *et al*., 2012). The study was approved by ethics committees at the seven study sites. Participants provided written, informed consent.

### Adverse event variables

The primary exposure of interest was the reported occurrence of an adverse event in childhood, defined as experiencing sexual abuse, other abuse (physical abuse, emotional abuse and neglect) or interpersonal loss (loss of a parent, sibling or close relative, parental separation or divorce). Only cases where the adverse event occurred during childhood (⩽18 years) and before the onset of psychotic illness were included.

Our adverse event variables were constructed from responses to a series of questions. Participants were initially asked the following questions: ‘Did your parents separate or divorce when you were a child?’; ‘Did you suffer the loss of a parent when you were a child?’; ‘Did you suffer the loss of a brother or sister when you were a child?’; ‘Did you suffer the loss of another close relative when you were a child?’ and ‘Were there any other distressing or traumatic events in your childhood?’. The age when each event happened was also recorded. These first four questions were used to construct a variable representing the presence or absence of interpersonal loss in childhood.

If participants reported any other distressing or traumatic events, then interviewers took notes of what had happened. These qualitative responses were coded into two main categories: sexual abuse and other types of abuse (defined as physical abuse, emotional abuse, neglect and other forms of abuse), based on Australian guidelines (Australian Institute of Health and Welfare, 2012). Further details about these variables are described elsewhere (Rosenman and Rodgers, 2004; Shah *et al*., 2014)

We constructed a variable representing the occurrence of any of these adverse events in childhood (0 *v*. ⩾1). We also separated them out into three variables: sexual abuse (yes, no), physical and emotional abuse and neglect (yes, no) and interpersonal loss (yes, no). This coding allowed for the possibility that an individual could experience more than one type of adverse event. Finally, to understand the effect of each type of adverse event in isolation (in particular, childhood adversity in the absence of abuse), we constructed three hierarchically coded variables. Reported sexual abuse trumped physical and emotional abuse and neglect and, physical and emotional abuse and neglect trumped interpersonal loss, such that individuals could only be counted once in the set of analyses.

### Outcome variables

The SHIP interview schedule included a number of embedded instruments (Morgan *et al*., 2012). Symptom profile and mental health diagnoses were determined using the Diagnostic Interview for Psychosis (DIP) (Castle *et al*., 2006). The DIP allows lifetime, previous year and previous 4 weeks’ ratings of symptoms; diagnoses can be assigned according to ICD-10 (World Health Organisation, 1992) and DSM-IV (American Psychiatric Association, 2000) criteria. The DIP contains selected interview questions and probes from the WHO Schedules for Clinical Assessment in Neuropsychiatry (Wing *et al*., 1990) mapped on the OPCRIT (McGuffin *et al*., 1991). DIP scores are fed into the computerised OPCRIT algorithm which produces diagnoses according to, in this case, the 10th edition of the International Classification of Disease (ICD-10) (World Health Organisation, 2015).

Using the DIP, we identified six symptoms or symptom clusters occurring over the lifetime. These were depressive symptoms, mania (elevated or irritable mood), self-reproach, delusions, hallucinations and subjective thought disorder. We used ratings of suicidal thoughts in the previous 4 weeks and self-harm in the previous 12 months. We also identified the presence of two additional symptoms and syndromes: anxiety in the previous 12 months using relevant Schedules for Clinical Assessment in Neuropsychiatry items (SCAN) (Wing *et al*., 1999; Bolton, 2010), and a count of the number of negative symptoms over the previous 12 months (using SCAN items of the Carpenter deficits syndrome) (Kirkpatrick *et al*., 1989).

We identified three diagnoses occurring over the lifetime, all using ICD-10 criteria (World Health Organisation, 1992). These were alcohol and other substance abuse and dependence (excluding cannabis), and diagnoses for affective psychosis and non-affective psychosis. Affective psychosis was defined as a diagnosis of bipolar disorder or mania (both in the presence of psychotic symptoms); non-affective psychosis by a diagnosis of schizophrenia, schizoaffective disorder or delusional disorders and other non-organic psychoses. Diagnoses of substance use and affective psychosis/non-affective psychosis were not mutually exclusive due to possible comorbidity.

Finally, we included five variables measuring psychosocial outcomes. Four of these were based on previous 12 months and were: behavioural and social functioning including role performance (assessed using the Personal and Social Performance Scale (PSP) (Morosini *et al*., 2000)); experience of victimisation (ABS Crime and Safety Survey) (Australian Bureau of Statistics, 2005); perpetration of crime or offending (extracted from the section on criminality within the Opiate Treatment Index (Darke *et al*., 1991)) and homelessness (Homelessness and Mental Health Survey (Robinson, 2003)). The fifth psychosocial outcome was the presence of a definite psychosocial stressor within 12 months of the first psychotic episode.

PSP scores range from 0 to 100, where 100 represents excellent functioning. The recognised cut-point of 70 or less is used to indicate poor functioning (scores above this value indicate the absence of disability or only mild difficulties). Experience of victimisation was measured using four items (number of times the participant was a victim of a break-in, theft, assault or was threatened with assault) and coded 0 *v*. ⩾1. Homelessness was measured by the number of homeless days in the previous year (coded 0 *v*. ⩾1 day). Perpetration of crime was measured using a positive response to the question: ‘in the last 12 months have you committed any crimes such as break and enter, shoplifting, dealing drugs, forging cheques, armed robbery or assault?’ Psychosocial stressors included potentially significant and/or threatening events that occurred during the 12 months preceding onset of psychosis such as: important problems with primary support groups, educational problems, occupational problems, housing problems, economic problems, legal problems and other problems.

### Statistical analysis

For our primary analysis we used logistic regression to model the effects of exposure to any adverse event on each of the 18 outcome variables. As a sensitivity analysis, we then repeated the analysis restricted to people with non-affective psychosis (‘sensitivity analysis 1’) and affective psychosis (‘sensitivity analysis 2’). For our secondary analysis we modelled separately the effects of exposure to each of the specific types of abuse or loss (sexual abuse, physical and emotional abuse and neglect, interpersonal loss) on the outcome variables. Finally and as a third sensitivity analysis, we modelled the effect of the three specific types of abuse or loss using the hierarchically coded variables whereby sexual abuse trumped physical and emotional abuse and neglect which trumped interpersonal loss (‘sensitivity analysis 3’). To account for potential confounding between the adverse event variables and the outcome variables, all models included age (18–25, 26–35, 36–45, 46–65), sex, currently married or in a *de facto* relationship or previously married (yes, no), presence of children or step children (yes, no), born in Australia (yes, no), highest qualification is a school-level qualification (yes, no), family history of mental illness (yes, no), socioeconomic status of the participant's residence (in quintiles) and lifetime cannabis use (yes, no). Socioeconomic status was based on the Index of Relative Socioeconomic Disadvantage (IRSED) (Australian Bureau of Statistics, 2011). We report the effect sizes for the adverse event exposures only.

## Results

### Characteristics of the sample

Among the 1825 participants, 14% were aged 18–25 years and 28% were aged 46–65 years (Table 1). Most were male (60%), born in Australia (82%), and had a family history of mental illness (58%). Forty-nine percent were married/in a *de facto* relationship or had been married and 40% had children or step children. Thirty-five percent lived in neighbourhoods of low socio-economic status (lowest two IRSED quintiles). Forty-seven percent met the ICD-10 criteria for schizophrenia, 16% schizoaffective disorder, 18% bipolar disorder or mania (both with psychotic symptoms), 9% severe depression without psychosis, 5% delusional disorder, 4% depressive psychosis, 1% screened positive for psychosis but did not meet full ICD-10 criteria for psychosis. About 9% were classified as dependent drinkers using the AUDIT and over two-thirds had a lifetime history of cannabis use. The prevalence of the outcomes ranged from 9% for homelessness (⩽12 months) to 87% reporting delusions (lifetime). Concerning functional outcomes other than homelessness, 39% reported victimisation, 11% had been involved in some type of crime and offending and 82% reported social dysfunction (all ⩽12 months).

**Table 1.** Characteristics of participants, *n*  = 1825

	Number	Percent
Covariates		
Age		
18–25	247	14
26–35	568	31
36–45	497	27
46–65	513	28
Male	1087	60
Currently married or has been married	891	49
Has children or step children	723	40
Born in Australia	1500	82
No tertiary qualification	919	51
Family history of mental illness	1060	58
Socioeconomic status (IRSED, quintile)		
1 (Poorest)	292	16
2	340	19
3	424	23
4	445	24
5 (Richest)	320	18
Lifetime cannabis use	1211	67
Exposure to childhood adverse event		
Experienced any adverse event	1466	80
Experienced sexual abuse	301	16
Experienced other types of abuse	305	17
Experienced interpersonal loss	1317	72
Outcome variables		
Depression (lifetime)	1469	80
Mania (lifetime)	794	44
Self-reproach (lifetime)	582	32
Delusions (lifetime)	1583	87
Hallucinations (lifetime)	1440	79
Subjective thought disorder (lifetime)	857	47
Suicidal thoughts (past 4 weeks)	210	12
Self-harm (past 12 months)	310	17
Anxiety (past 12 months)	1145	63
Negative syndrome (past 12 months)	1291	71
Alcohol and other substance dependence, excluding cannabis (lifetime, ICD-10)	1071	59
Affective psychosis (lifetime, ICD-10)	400	22
Non-affective psychosis (lifetime, ICD-10)	1242	68
Social dysfunction (past 12 months)	1502	82
Victimisation (past 12 months)	717	39
Crime/offending (past 12 months)	201	11
Homelessness (past 12 months)	159	9
Definite psychosocial stressor within 12 months of first episode	1147	63

*Note*: Numbers may not sum to 1825 due to missing data. IRSED is Index of Relative Socioeconomic Disadvantage.

### Exposure to adverse events

Eighty percent of participants had experienced an adverse event in childhood – 16% had experienced sexual abuse, 17% had experienced other forms of abuse and 72% had experienced interpersonal loss. Parental separation or divorce was the most common interpersonal loss (40%).

### Outcomes associated with any adverse event

The occurrence of any adverse childhood event was associated with increased odds of 12/18 symptoms, diagnoses and psychosocial outcomes (Table 2). For instance, those who had experienced a childhood adverse event had 1.68 times higher odds of having depression compared with those who had not experienced an adverse event. The largest effect sizes were for homelessness (odds ratio (OR) = 2.82), participation in crime and offending in the previous 12 months (OR = 1.95) and suicidal thoughts in the previous 4 weeks (OR = 1.90). Of the three diagnoses examined, the only association observed was for alcohol and other substance dependence (OR = 1.39).

**Table 2.** Logistic regression models estimating the effect of any adverse event on 18 variables measuring symptoms, diagnoses and functioning (primary and sensitivity analyses)

	Primary analysis		Sensitivity analysis 1		Sensitivity analysis 2	
	*n* = 1793		Non-affective psychosis		Affective psychosis	
			*n* = 1222		*n* = 391	
Outcome	AOR (95% CI)	*p*-value	AOR (95% CI)	*p*-value	AOR (95% CI)	*p*-value
Symptom and syndrome profile						
Depression (lifetime)	**1.68** (**1.26–2.24)**	**<0**.**001**	**1.61** (**1.17–2.20)**	**0**.**003**	1.40 (0.50–3.95)	0.522
Mania (lifetime)	1.09 (0.85–1.41)	0.486	1.31 (0.95–1.81)	0.100	**0.28 (0.10–0.76)**	**0.012**
Self-reproach (lifetime)	1.21 (0.92–1.60)	0.166	1.08 (0.77–1.53)	0.653	**2.06 (1.15–3.68)**	**0.015**
Delusions (lifetime)	0.98 (0.68–1.41)	0.895	1.25 (0.68–2.31)	0.476	1.05 (0.49–2.26)	0.893
Hallucinations (lifetime)	**1.40 (1.05–1.85)**	**0.020**	**1.54 (1.04–2.27)**	**0.030**	1.76 (0.99–3.13)	0.055
Subjective thought disorder (lifetime)	**1.49 (1.16–1.91)**	**0.002**	**1.64 (1.22–2.21)**	**0.001**	1.31 (0.71–2.41)	0.391
Suicidal thoughts (past 4 weeks)	**1.90 (1.20–3.01)**	**0.006**	**2.01 (1.09–3.71)**	**0.025**	1.43 (0.65–3.15)	0.379
Self-harm (past 12 months)	**1.63 (1.11–2.40)**	**0.013**	1.35 (0.82–2.20)	0.235	2.26 (0.98–5.22)	0.055
Anxiety (past 12 months)	**1.54 (1.20–1.97)**	**0.001**	**1.48 (1.10–1.98)**	**0.009**	1.34 (0.73–2.45)	0.347
Negative syndrome (past 12 months)	0.98 (0.75–1.29)	0.903	0.80 (0.56–1.12)	0.191	1.41 (0.78–2.54)	0.255
Diagnosis						
Alcohol and other substance dependence excluding cannabis (lifetime)	**1.39 (1.05–1.83)**	**0.020**	1.26 (0.90–1.76)	0.184	1.62 (0.87–3.02)	0.126
Affective psychosis (lifetime)	1.06 (0.78–1.45)	0.703	–	–	–	–
Non-affective psychosis (lifetime)	0.88 (0.66–1.16)	0.351	–	–	–	–
Functioning						
Social dysfunction (past 12 months)	**1.51 (1.10–2.07)**	**0.010**	1.40 (0.93–2.10)	0.106	1.55 (0.80–3.00)	0.191
Victimisation (past 12 months)	**1.56 (1.20–2.03)**	**0.001**	**1.53 (1.12–2.10)**	**0.008**	1.14 (0.63–2.05)	0.663
Crime/offending (past 12 months)	**1.95 (1.18–3.21)**	**0.009**	1.60 (0.89–2.87)	0.117	2.90 (0.98–8.55)	0.054
Homelessness (past 12 months)	**2.82 (1.52–5.22)**	**0.001**	**2.35 (1.18–4.67)**	**0.015**	3.28 (0.71–15.15)	0.128
Definite psychosocial stressor within 12 months of first episode	**1.74 (1.34–2.24)**	**<0.001**	**2.00 (1.47–2.72)**	**<0.001**	**1.87 (1.01–3.43)**	**0.045**

Significant values are in bold.

*Note*: Models adjusted for age, sex, marital status, parental status, born in Australia, highest qualification obtained, family history of mental illness, socioeconomic status and lifetime cannabis use.

In a sensitivity analysis where the sample was restricted to people with non-affective psychosis, these findings largely held, with any adverse event in childhood still associated with eight outcomes. Most ORs were of similar magnitude to the primary analysis. In a second sensitivity analysis restricted to people with affective psychosis, an adverse event in childhood was associated with only three outcomes. Any adverse event in childhood was associated with lower odds of mania (OR = 0.28) and higher odds of self-reproach (OR = 2.06) and a definite psychosocial stressor within 12 months of first episode (OR = 1.87). Some large ORs were non-significant, suggesting that the reduced sample size used for these analyses may have been underpowered to detect effects.

### Outcomes associated with specific adverse events

Table 3 shows the association between three specific adverse events and the outcomes under investigation. Sexual abuse in childhood predicted 10/18 outcomes. Other forms of abuse (physical abuse, emotional abuse and neglect) were associated with 13 outcomes, and experiencing interpersonal loss (loss of a parent, loss of a sibling, loss of a close relative, parental separation or divorce) was associated with four outcomes. Common across all types of adverse events was the association with depression, anxiety and a definite psychosocial stressor within 12 months of first episode.

**Table 3.** Secondary analysis: logistic regression models estimating the effect of sexual abuse, other abuse and interpersonal loss on 18 variables measuring symptoms, diagnoses and measures of functioning, *n*  =  1793

	Sexual abuse		Other abuse		Interpersonal loss	
Outcomes	AOR (95% CI)	*p*-value	AOR (95% CI)	*p*-value	AOR (95% CI)	*p*-value
Symptom and symptom clusters						
Depression (lifetime)	2.16 (1.38–3.37)	**0**.**001**	**1.53** (**1.05–2.22)**	**0**.**027**	**1.41** (**1.08–1.83)**	**0**.**012**
Mania (lifetime)	1.25 (0.96–1.64)	0.101	0.97 (0.74–1.25)	0.790	1.03 (0.83–1.29)	0.780
Self-reproach (lifetime)	1.64 (1.25–2.15)	**<0.001**	**1.54 (1.18–2.01)**	**0.001**	1.00 (0.79–1.26)	0.999
Delusions (lifetime)	1.04 (0.71–1.52)	0.833	1.39 (0.92–2.09)	0.116	0.92 (0.66–1.27)	0.600
Hallucinations (lifetime)	1.53 (1.08–2.18)	**0.017**	**1.45 (1.03–2.03)**	**0.032**	1.25 (0.97–1.61)	0.087
Subjective thought disorder (lifetime)	1.64 (1.25–2.15)	**<0.001**	**1.33 (1.03–1.73)**	**0.029**	1.14 (0.91–1.42)	0.248
Suicidal thoughts (last 4 weeks)	1.56 (1.08–2.26)	**0.018**	**1.72 (1.21–2.44)**	**0.003**	1.25 (0.88–1.78)	0.209
Self-harm (past 12 months)	1.79 (1.31–2.45)	**<0.001**	**1.55 (1.13–2.12)**	**0.006**	1.27 (0.93–1.72)	0.131
Anxiety (past 12 months)	1.56 (1.16–2.11)	**0.003**	**1.64 (1.23–2.18)**	**0.001**	**1.26 (1.01–1.58)**	**0.037**
Negative syndrome (past 12 months)	1.39 (1.03–1.88)	**0.031**	**1.40 (1.04–1.87)**	**0.027**	1.01 (0.80–1.28)	0.926
Diagnosis						
Alcohol and other substance dependence excluding cannabis (lifetime)	1.17 (0.86–1.59)	0.307	1.21 (0.90–1.63)	0.212	1.16 (0.91–1.48)	0.225
Affective psychosis (lifetime)	1.22 (0.90–1.65)	0.197	1.13 (0.83–1.53)	0.439	0.86 (0.66–1.11)	0.254
Non-affective psychosis (lifetime)	0.96 (0.72–1.27)	0.777	0.98 (0.74–1.29)	0.883	0.98 (0.78–1.25)	0.892
Functioning						
Social dysfunction (past 12 months)	1.40 (0.98–2.00)	0.066	**1.48 (1.03–2.14)**	**0.036**	1.21 (0.91–1.60)	0.193
Victimisation (past 12 months)	1.48 (1.14–1.94)	**0.004**	**1.80 (1.39–2.32)**	**<0.001**	1.19 (0.95–1.49)	0.121
Crime/offending (past 12 months)	1.44 (0.95–2.18)	0.084	**1.84 (1.28–2.66)**	**0.001**	1.31 (0.89–1.92)	0.173
Homelessness (past 12 months)	1.00 (0.62–1.62)	0.994	**1.92 (1.29–2.87)**	**0.001**	**1.91 (1.20–3.02)**	**0.006**
Definite psychosocial stressor within 12 months of first episode	1.66 (1.21–2.26)	**0.001**	**1.71 (1.27–2.30)**	**<0.001**	**1.26 (1.00–1.58)**	**0.049**

Significant values are in bold.

*Note*: Models adjusted for age, sex, marital status, parental status, born in Australia, highest qualification obtained, family history of mental illness, socioeconomic status and lifetime cannabis use.

The relationship between sexual abuse in childhood and the outcome variables was strongest for depression (OR = 2.16), self-harm (OR = 1.79) and a definite psychosocial stressor within 12 months of first episode (OR = 1.66). The effect of other forms of abuse was strongest for homelessness (OR = 1.92), participation in crime and offending (OR = 1.84) and victimisation (OR = 1.80). Interpersonal loss in childhood was associated with homelessness (OR = 1.91), depression (OR = 1.41), anxiety (OR = 1.26) and definite psychosocial stressor within 12 months of first episode (OR = 1.26).

In the third sensitivity analysis where abuse and loss were coded hierarchically, the results for sexual abuse and other forms of abuse were broadly similar to that reported in the non-hierarchically coded analysis (Table 4). However, a different picture emerged for interpersonal loss. This form of adversity (in the absence of the other two) was associated with lower odds of self-reproach (OR = 0.70), negative syndrome (OR = 0.75) and victimisation (OR = 0.82).

**Table 4.** Sensitivity analysis 3: logistic regression models estimating the effect of sexual abuse, other abuse and interpersonal loss (hierarchically coded) on 18 variables measuring symptoms, diagnoses and measures of functioning, *n*  =  1793

	Sexual abuse		Other abuse		Interpersonal loss	
Outcomes	AOR (95% CI)	*p*-value	AOR (95% CI)	*p*-value	AOR (95% CI)	*p*-value
Symptom and symptom clusters						
Depression (lifetime)	**2.16** (**1.38–3.37)**	**0**.**001**	1.47 (0.99–2.19)	0.058	0.92 (0.72–1.18)	0.504
Mania (lifetime)	1.25 (0.96–1.64)	0.101	0.89 (0.67–1.18)	0.411	0.99 (0.82–1.21)	0.948
Self-reproach (lifetime)	**1.64 (1.25–2.15)**	**<0.001**	**1.44 (1.09–1.92)**	**0.011**	**0.70 (0.57–0.86)**	**0.001**
Delusions (lifetime)	1.04 (0.71–1.52)	0.833	1.40 (0.90–2.19)	0.137	0.83 (0.62–1.10)	0.195
Hallucinations (lifetime)	**1.53 (1.08–2.18)**	**0.017**	1.42 (0.99–2.04)	0.056	0.86 (0.68–1.09)	0.221
Subjective thought disorder (lifetime)	**1.64 (1.25–2.15)**	**<0.001**	**1.34 (1.01–1.76)**	**0.040**	0.85 (0.70–1.03)	0.105
Suicidal thoughts (last 4 weeks)	**1.56 (1.08–2.26)**	**0.018**	1.43 (0.97–2.10)	0.071	0.87 (0.65–1.17)	0.351
Self-harm (past 12 months)	**1.79 (1.31–2.45)**	**<0.001**	1.22 (0.86–1.73)	0.268	0.80 (0.62–1.03)	0.088
Anxiety (past 12 months)	**1.56 (1.16–2.11)**	**0.003**	**1.56 (1.15–2.11)**	**0.004**	0.87 (0.72–1.07)	0.185
Negative syndrome (past 12 months)	**1.39 (1.03–1.88)**	**0.031**	1.24 (0.90–1.69)	0.184	**0.75 (0.61–0.93)**	**0.009**
Diagnosis						
Alcohol and other substance dependence excluding cannabis (lifetime)	1.17 (0.86–1.59)	0.307	1.23 (0.89–1.69)	0.212	1.03 (0.82–1.28)	0.815
Affective psychosis (lifetime)	1.22 (0.90–1.65)	0.197	1.17 (0.85–1.62)	0.334	0.85 (0.67–1.07)	0.167
Non-affective psychosis (lifetime)	0.96 (0.72–1.27)	0.777	1.00 (0.74–1.35)	0.998	0.95 (0.77–1.17)	0.609
Functioning						
Social dysfunction (past 12 months)	1.40 (0.98–2.00)	0.066	1.42 (0.95–2.11)	0.083	0.92 (0.71–1.19)	0.534
Victimisation (past 12 months)	**1.48 (1.14–1.94)**	**0.004**	**1.64 (1.25–2.16)**	**<0.001**	**0.82 (0.67–0.99)**	**0.043**
Crime/offending (past 12 months)	1.44 (0.95–2.18)	0.084	**1.67 (1.12–2.48)**	**0.011**	0.84 (0.61–1.15)	0.265
Homelessness (past 12 months)	1.00 (0.62–1.62)	0.994	**1.84 (1.20–2.81)**	**0.005**	1.11 (0.79–1.57)	0.542
Definite psychosocial stressor within 12 months of first episode	**1.66 (1.21–2.26)**	**0.001**	**1.57 (1.15–2.14)**	**0.005**	0.91 (0.74–1.12)	0.390

Significant values are in bold.

*Note*: Models adjusted for age, sex, marital status, parental status, born in Australia, highest qualification obtained, family history of mental illness, socioeconomic status and lifetime cannabis use.

## Discussion

This study is unique in using a large representative population of people with psychosis to examine the prevalence of diverse adverse events in childhood and their relationship with symptomatic and psychosocial outcomes. It adds to previous research by collecting systematic data on a wide range of clinical and psychosocial outcomes, some previously unexamined in this context (including homelessness and the presence of a definite psychosocial stressor within 12 months of illness onset).

Four in five Australians living with psychosis reported experiencing some form of adverse event in childhood and approximately one in six reported exposure to childhood sexual abuse on at least one occasion before 18 years of age. However, since ascertainment of abuse histories by retrospective rather than prospective methods (Hooven *et al*., 2012; Varese *et al*., 2012), or by face-to-face interviews compared with confidential self-report measures (Bendall *et al*., 2008), tends to underestimate this type of abuse, we expect this figure may be greater. Our reported prevalence of childhood sexual abuse within this population falls within previously reported ranges (e.g. Gustafson and Sarwer, 2004; Casey and Nurius, 2005; Messman-Moore *et al*., 2010). These findings support previously reported increased rates of childhood adversity in people with diagnoses of mental illness when compared to the general population (Morgan and Fisher, 2007; Cutajar *et al*., 2010; McCabe *et al*., 2012; Mauritz *et al*., 2013).

The other key finding is that more than one type of adversity appears to be associated with the development of psychosis (van Nierop *et al*., 2014). Our results also suggest that childhood interpersonal loss, occurring in the absence of other forms of abuse, contributes to less severe symptom and functioning impacts within the population of people with psychosis.

A number of functional outcomes were strongly associated with physical and emotional abuse and neglect, especially victimisation, offending and homelessness, adding to a growing body of evidence about the functional sequelae of childhood adversity (e.g. Cantor-Graae, 2007; Rosenberg *et al*., 2007; Gil *et al*., 2009). As in the Gil study, the strongest associations were with reported abuse other than sexual abuse. This highlights the potentially pervasive negative impact of all forms of childhood adversity in this sub-population and the likely contribution of adverse childhood experiences to psychosocial disability and complex needs among people with psychosis (Harvey *et al*., 2016).

### Subsequent adversities

Our findings demonstrate that the association between childhood adversity and subsequent adversity previously recognised within the general population (e.g. Briere and Elliott, 2003; Casey and Nurius, 2005) is also evident in people with psychosis. For all participants, exposure to a definite psychosocial stressor within the 12 months preceding illness onset was high (63%) as was recent victimisation (39%). Those participants reporting any form of childhood adversity (and those who had experienced sexual abuse or other forms of abuse) had a higher rate of reporting a definite psychosocial stressor within 12 months of illness onset and also recent victimisation. These findings are consistent with the possibility that perceived social threat is a key risk factor for psychosis (van Nierop *et al*., 2014). This also supports cognitive models of psychosis that suggest particular psychotic symptoms may be best conceptualised as dimensional such that expression of these symptoms is linked to exposure to cumulative adverse events beginning in childhood. This may also be associated with increased stress reactivity later in life (Perry *et al*., 1995; Garety *et al*., 2007; Bolton, 2010). It is plausible that subsequent experiences of adverse events could be an important contributor in elevating cortisol levels which in turn may affect the severity of depressive and anxiety symptoms and increase the risk of developing psychosis (Garety *et al*., 2007). This theory is also supported by our finding that depression and anxiety are associated with exposure to any type of childhood adversity.

### Positive psychotic symptoms

We found associations between any childhood adversity and positive psychotic symptoms of hallucinations and subjective thought disorder but not delusions. Hallucinations were strongly associated with childhood sexual abuse and we found a relationship between subjective thought disorder and all forms of childhood abuse, as reported elsewhere (Schenkel *et al*., 2005; McCabe *et al*., 2012; Shah *et al*., 2014). While some studies have found a relationship between child abuse and delusions (Scott *et al*., 2007; van Nierop *et al*., 2014), most find a stronger association between child abuse and hallucinations. Such studies also show a dose–response relationship between the number and types of traumatic events and delusional experiences, such that a combination of child abuse and adult abuse may be a strong predictor of delusional experiences (Read *et al*., 2003; Scott *et al*., 2007). In line with other studies, we found an association between any form of childhood adversity and alcohol and other substance dependence. There were no associations between abuse and interpersonal loss and lifetime diagnoses of either affective psychosis or non-affective psychosis, consistent with a number of other reports of a lack of diagnostic specificity (Matheson *et al*., 2013; Shah *et al*., 2014; van Nierop *et al*., 2015). In analyses restricted to people with affective psychosis, we found a different pattern of associations to that observed for people with non-affective psychosis. However, the sample of people with affective psychosis was small making interpretation difficult. Future research could explore this issue further. Our results suggest that there may be a link between event type and the increased likelihood of particular symptomatic responses. Previous studies have also found a dose–response relationship between the severity of sexual abuse in childhood and risk of hallucinations in adulthood (Shevlin *et al*., 2007; Bentall *et al*., 2012), however this was not within the scope of our study.

### Negative schematic belief systems

Those who had experienced sexual abuse had a higher likelihood of depression, more than twice that of those who had not experienced abuse. Further and consistent with the literature, self-harm and suicidal thoughts were associated with childhood abuse (Schenkel *et al*., 2005; Rosenberg *et al*., 2007; Read *et al*., 2008; Conus *et al*., 2009; Hooven *et al*., 2012; Shah *et al*., 2014; van Nierop *et al*., 2014). We also found associations between abuse experiences and self-reproach which add to existing theories regarding emotional sequelae of abuse, including negative schematic beliefs and self-appraisals, and their proposed mediating role in facilitating or maintaining psychosis (Garety *et al*., 2007; Gracie *et al*., 2007). Likewise, these findings suggest avenues for study of conceivable causal pathways and tailored psychological interventions in this population (Mueser *et al*., 2008).

### Limitations

The cross-sectional design is a limitation of this study as is the fact that all the individuals who entered our study had a diagnosis of psychosis; we were therefore unable to compare those who had experienced childhood trauma with people who had similar experiences in the general population. Recall bias associated with retrospective reporting of adversity is possible, although retrospective methods tend to bias towards under-reporting rather than over-reporting (Varese *et al*., 2012). Although carefully operationalised according to recognised definitions (Shah *et al*., 2014), our abuse types were based on a single question. The presence of these was determined from a single open-ended question about other distressing or traumatic events in childhood, with detailed notes taken and responses coded into categories. However, this may have further contributed to underreporting. Further, we recognise the limitations of using dichotomous variables meaning some of the detail of the experience is lost. Finally, our coding scheme meant that we did not distinguish between the occurrence of physical and emotional abuse and neglect (all were coded as other abuse). Understanding how specific types of abuse and neglect in childhood may contribute to the development of mental and psychosocial impairment in adulthood would be a useful focus of future research.

## Conclusions

In our sample of people living with psychosis, adverse events in childhood, especially interpersonal loss, were common and suggest a need for appropriate and targeted prevention. Sexual abuse and other types of abuse were less common but may lead to elevated risk of poorer mental health and poorer psychosocial outcomes in adulthood relative to other people with psychosis. An implication of these findings is that mental health professionals should be trained to make routine and sensitive enquiries about adverse childhood events since these may have prognostic implications for mental health outcomes. Further, mental health professionals and researchers should develop and apply the evidence base for trauma-focussed interventions. Longitudinal research designs are warranted to provide stronger evidence about the risk of developing psychosis following adverse events in childhood and the potential role of subsequent psychosocial stressors. These designs should also gather more details about the exposure (e.g. age of occurrence, frequency of adverse event types and victim's relationship to perpetrator), whether disclosure occurred and what response occurred to any disclosure, in order to further improve our understanding of relationships between childhood adversity and psychosis.

## Data

The 2010 Survey of High Impact Psychosis is available for approved researchers to access. Contact Professor Vera Morgan, Head: Neuropsychiatric Epidemiology Research Unit, School of Population and Global Health, University of Western Australia, email: vera.morgan@uwa.edu.au.
